# Intermediate risk prostate tumors contain lethal subtypes

**DOI:** 10.3389/fruro.2024.1487873

**Published:** 2025-01-15

**Authors:** William L. Harryman, James P. Hinton, Rafael Sainz, Jaime M. C. Gard, John M. Ryniawec, Gregory C. Rogers, Noel A. Warfel, Beatrice S. Knudsen, Raymond B. Nagle, Juan J. Chipollini, Benjamin R. Lee, Belinda L. Sun, Anne E. Cress

**Affiliations:** ^1^ University of Arizona Cancer Center, Tucson, AZ, United States; ^2^ Department of Cellular and Molecular Medicine, University of Arizona, Tucson, AZ, United States; ^3^ Professor of Pathology and Biomedical Sciences, Huntsman Cancer Institute, University of Utah, Salt Lake City, UT, United States; ^4^ Department of Urology, University of Arizona College of Medicine, Tucson, AZ, United States; ^5^ Department of Pathology, University of Arizona College of Medicine, Tucson, AZ, United States

**Keywords:** prostate cancer, cribriform, intraductal carcinoma, gleason grade, intermediate risk, biomarkers

## Abstract

In 2024, prostate cancer (PCa) remains the most common non-skin cancer in males within the United States, with an estimated 299,010 new cases, the highest increase incident trend rate (3.8%) of all cancers, and one of the eight deadliest. PCa cases are projected to double from 1.8 million to 2.9 million per year between 2020 and 2040. According to the National Comprehensive Cancer Network (NCCN) treatment guidelines, most cases (65%) are intermediate risk (Gleason sum score <7 [3 + 4, 4 + 3], prostate organ-confined, and PSA < 20) with treatment options limited to active surveillance, external beam radiation, and/or surgery to prevent metastasis in the long term (>10 years). It is increasingly recognized that the two most common subtypes of intermediate risk PCa are cribriform architecture (CA) and intraductal carcinoma of the prostate (IDC-P), which can occur together, and both are associated with increased metastatic risk, biochemical recurrence, and disease-specific mortality. Both subtypes display hypoxia, genomic instability, and are identified as Gleason 4 in pathology reports. However, since false negatives are common (up to 50%) in these subtypes on biopsy, more research is needed to reliably detect these subtypes that have an increased risk for invasive disease. We note that even with mpMRI-guided biopsies, the sensitivity is 54% for cribriform architecture and only 37% for IDC-P. The presence of these PCa subtypes in biopsy or radical prostatectomy (RP) tissue can exclude patients from active surveillance and from designation as intermediate risk disease, further underscoring the need for increased molecular understanding of these subtypes for diagnostic purposes. Understanding the heterogeneity of intermediate risk primary PCa phenotypes, using computational pathology approaches to evaluate the fixed biopsy specimen, or video microscopy of the surgical specimen with AI-driven analysis is now achievable. New research associating the resulting phenotypes with the different therapeutic choices and vulnerabilities will likely prevent extracapsular extension, the definition of high-risk disease, and upstaging of the final pathologic stage.

## 1 Introduction

Localized prostate cancer (PCa) frequently harbors several spatially distinct tumors containing considerable inter- and intra-tumoral heterogeneity, producing genetically diverse clones that develop in the hypoxic peripheral zone of the prostate (1, 2). Because PCa proliferates slowly, with Gleason sum score ≤ 7 tumors having a low mitotic index of approximately 2.7 to 4.3% (3), the hypoxic microenvironment of the tumor due to decreased blood flow in the prostate is attributed to aging rather than a high tumor burden outstripping the blood supply. Prostatic adenocarcinoma, comprised of both acinar adenocarcinoma and, to a much lesser extent, ductal adenocarcinoma, is the most common tumor type of localized PCa, accounting for roughly 95% of diagnoses (4). Clinically, diagnosis and prognosis is largely based on a combination of histological criteria, including Gleason score, prostate-specific antigen levels, and TNM classification (5). The unmet clinical need, in addition to the presence of these diagnostic tools, is to identify which patients harbor tumors that are not indolent but will progress to become aggressive disease even after curative therapies such as prostatectomy (5).

### 1.1 Prevalence

1 in 8 US men will be diagnosed with prostate cancer at some point in their lives, with the 5-year survival rate at 99% for organ-confined disease; however, if the tumor penetrates the pseudo-capsule or escapes the gland through perineural invasion and becomes metastatic, the 5-year survival rate falls to ~37% (6). Recent projections indicate that the annual number of new cases of PCa will nearly double from 1.8 million to 2.9 million between 2020 and 2040 (7). In 2023, approximately 34,700 men died in the U.S. as a result of metastatic PCa that became resistant to typical treatment (8). High risk disease is defined as stage T3a, Gleason grade ≥7 [4 + 3], PSA >20ng/ml, with androgen deprivation therapy (ADT) as the standard of care (9). The curative intent is extended by use of 2nd-generation androgen receptor signaling inhibitors (ARSi) like abiraterone acetate and enzalutamide, to produce “complete androgen blockade” (10). However, despite inducing temporary remission, efforts to block all androgens eventually fail due to the emergence of a physiological bypass to include, in part, androgen receptor (AR) splice variants (such as AR-V7 (11)) made by the tumor or alternative sources of androgen supplied by the gut microbiota (12). The resulting castration-resistant prostate cancer (CRPC) is no longer treatable with current first-line therapies (13, 14). An unmet clinical need is the ability to identify which low and intermediate risk tumors can be assigned to Active Surveillance (AS) (favorable risk) and those that require treatment (unfavorable risk).

### 1.2 Intermediate risk prostate cancer

Intermediate favorable risk PCa (**Table 1**) is defined by the National Comprehensive Cancer Network (NCCN) as having all the following: no high-risk or very high-risk group features; one of the intermediate risk factors [cT2b– cT2c, Grade Group (GG) 1 or 2, PSA 10–20 ng/mL], and <50% biopsy cores positive. The standard of care for these patients is either active surveillance or primary treatment according to the NCCN guidelines (9). Hence, intermediate favorable risk tumors are either untreated (active surveillance) or treated (**Table 2**) with either radical prostatectomy and/or radiotherapy, with the addition of adjuvant therapy if pathology reports indicate the presence of risk factors missed in biopsy or detectable and rising PSA >0.1 ng/mL (9). In a study of nearly a thousand men with intermediate risk disease at diagnosis enrolled in an active surveillance trial, 44% of the men who eventually developed metastatic disease presented with Gleason 3 + 4 disease, while 26% presented with clinical features of very low risk prostate cancer (15); for this cohort, 10- and 15-year overall survival (OS) rates were 80% and 62%, respectively (16). In a new study, Liss et al. (2024), found that 67% of men given a GG1 diagnosis at biopsy were later upgraded following radical prostatectomy to GG2 or higher (17). The authors state that none of the assessed genetic risk factors were predictive of upgrading, including polygenic risk scores for prostate cancer diagnosis (17). Therefore, advances in our understanding of intermediate risk disease are necessary to identify those patients who will progress to high-risk PCa.

**Table 1:** NCCN initial risk stratification & staging for clinically localized disease.

	Favorable	Unfavorable
Life expectancy	More than 10 years	
High-risk features	No high or very-high-risk group features	
PSA	≤10 ng/mL	10–20 ng/mL
Intermediate risk features (from GG, biopsy, stage)	1 intermediate risk feature	2 or 3 intermediate risk features
Grade Group	1 or 2	3
Biopsy	<50% biopsy cores positive (e.g., <6 of 12 cores)	≥50% biopsy cores positive (e.g., ≥6 of 12 cores)
Clinical stage	<cT2b	cT2b–cT2c

**Table 2:** Treatment approaches for NCCN favorable & unfavorable intermediate risk PCa.

	Favorable	Unfavorable
Life expectancy	More than 10 years	
First option treatment	Active surveillance	Radical prostatectomy AND pelvic lymph node dissection
Second line treatment	Brachytherapy OR External Beam Radiation Therapy	External Beam Radiation Therapy w/ADT (4-6 months)
Aggressive treatment	Radical prostatectomy +/- pelvic lymph node dissection	External Beam Radiation Therapy AND Brachytherapy +/- ADT (4-6 months)

Intermediate risk disease commonly contains aggressive PCa subtypes that are associated with higher cancer-specific mortality but can be missed during biopsy or are not considered during pathological scoring (18, 19). Furthermore, genomic studies have revealed that metastases are monoclonal in nature and can be tracked back to these aggressive subclones in the primary tumor (20, 21). However, how these subtypes emerge in intermediate disease is poorly understood. While the presence of these subtypes is associated with higher cancer-specific mortality, their contribution to disease progression and metastasis are poorly understood. Model studies of extracapsular extension (ECE) show that heterotypic mixtures of tumor subtypes provide an advantage for PCa invasive clusters of cells to move through contractile muscle and seed within bone metastatic sites (22). Here we present a narrative review of aggressive PCa subtypes that can be found in intermediate risk tumors. This review focuses on cribriform architecture and intraductal carcinoma of the prostate, as these two features are often considered to be uncommon despite a considerable body of recent work indicating that they are not rare at all, but are frequently difficult to detect and missed in biopsy or imaging at the diagnostic stage.

## 2 Cribriform architecture and intraductal carcinoma of the prostate

**Figure 1:**
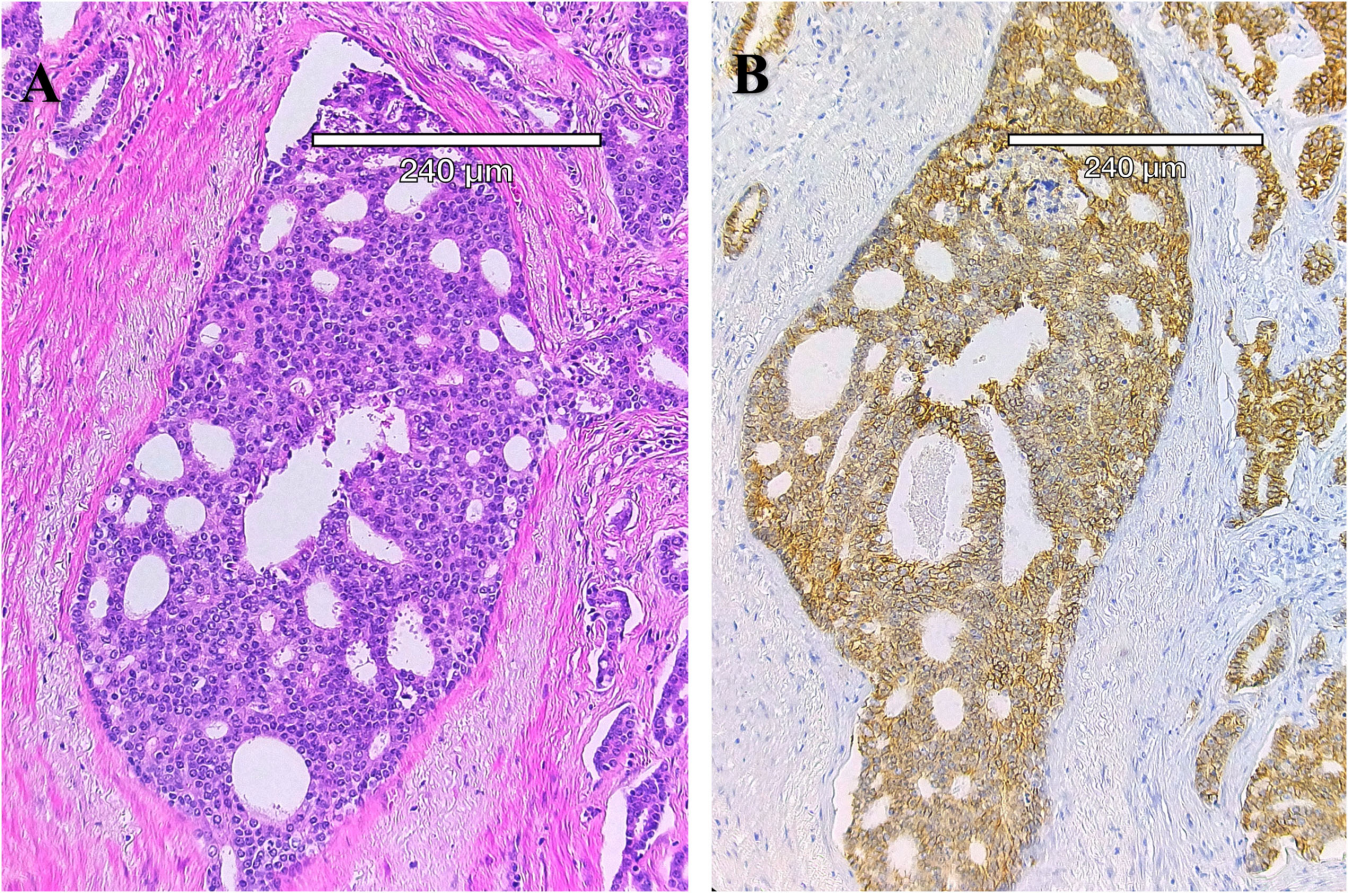
Cribriform architecture in a human prostatectomy tumor sample. **(A)** Hematoxylin and Eosin- stain of the gland shows the lack of basal cells and the enlarged nucleoli, as well as the “Swiss cheese” appearance. **(B)** Immunohistochemistry (E-cadherin, brown) with cell-cell junctions in this PCa subtype. Note the “glands within glands” appearance that partially defines the cribriform architecture, as well as the lack of basal cells around the gland. Despite the absence of basal cells, this subtype remains organized into clusters of cells.

**Figure 2:**
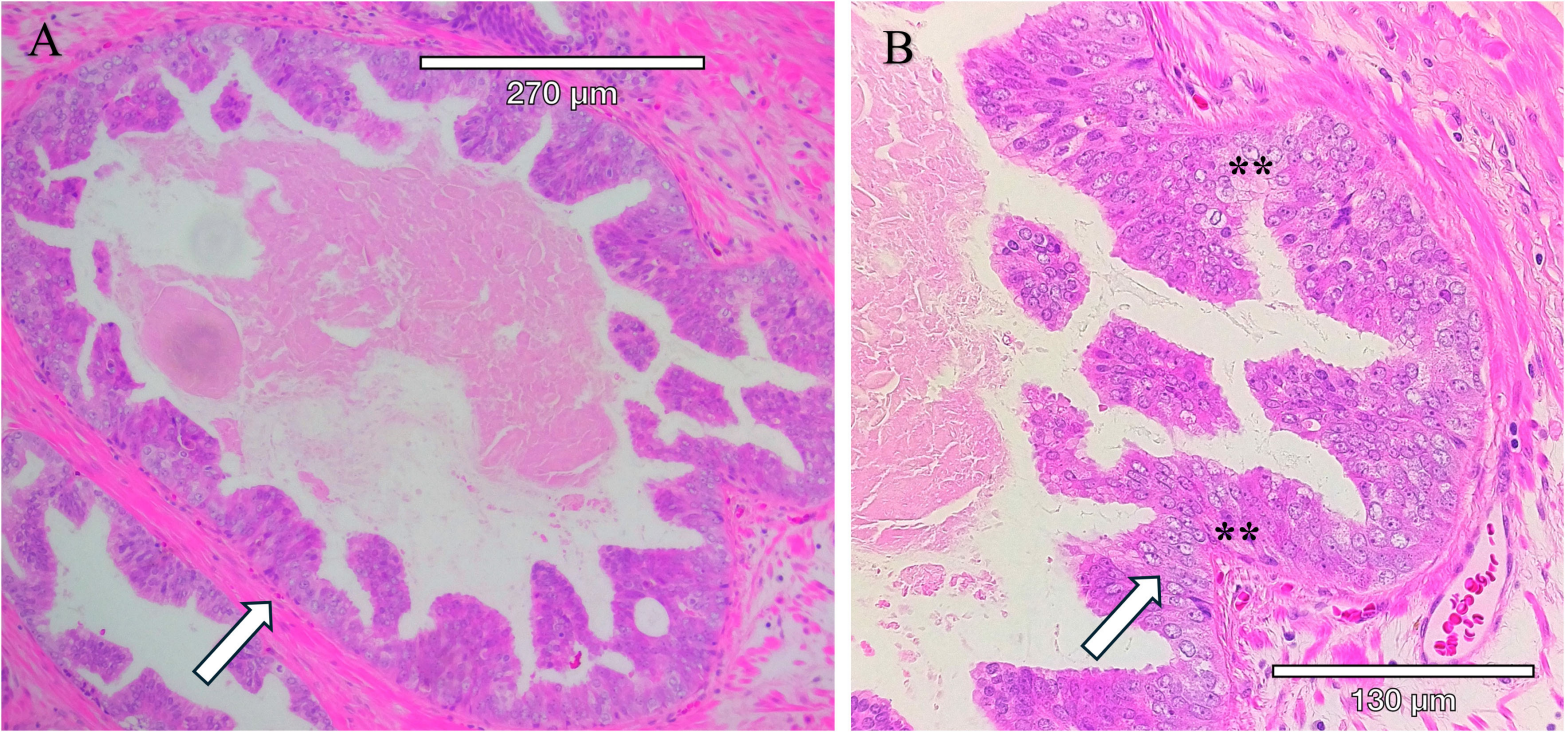
Intraductal carcinoma of the prostate present within a radical prostatectomy sample. IDC-P is characterized by the proliferation of malignant cells within existing prostatic ducts and acini and solid or dense cribriform architecture, marked nuclear atypia with nucleomegaly, and nonfocal comedonecrosis. **(A)** Hematoxylin and Eosin-stained (H&E) section. **(B)** Higher magnification of H&E-stained section with arrows pointing to tumor within the circumscribed gland. Tumor cells within the gland have enlarged nucleoli, an increased nuclear to cytoplasmic ratio, condensed heterochromatin, and apoptotic cells (**).

**Figure 3:**
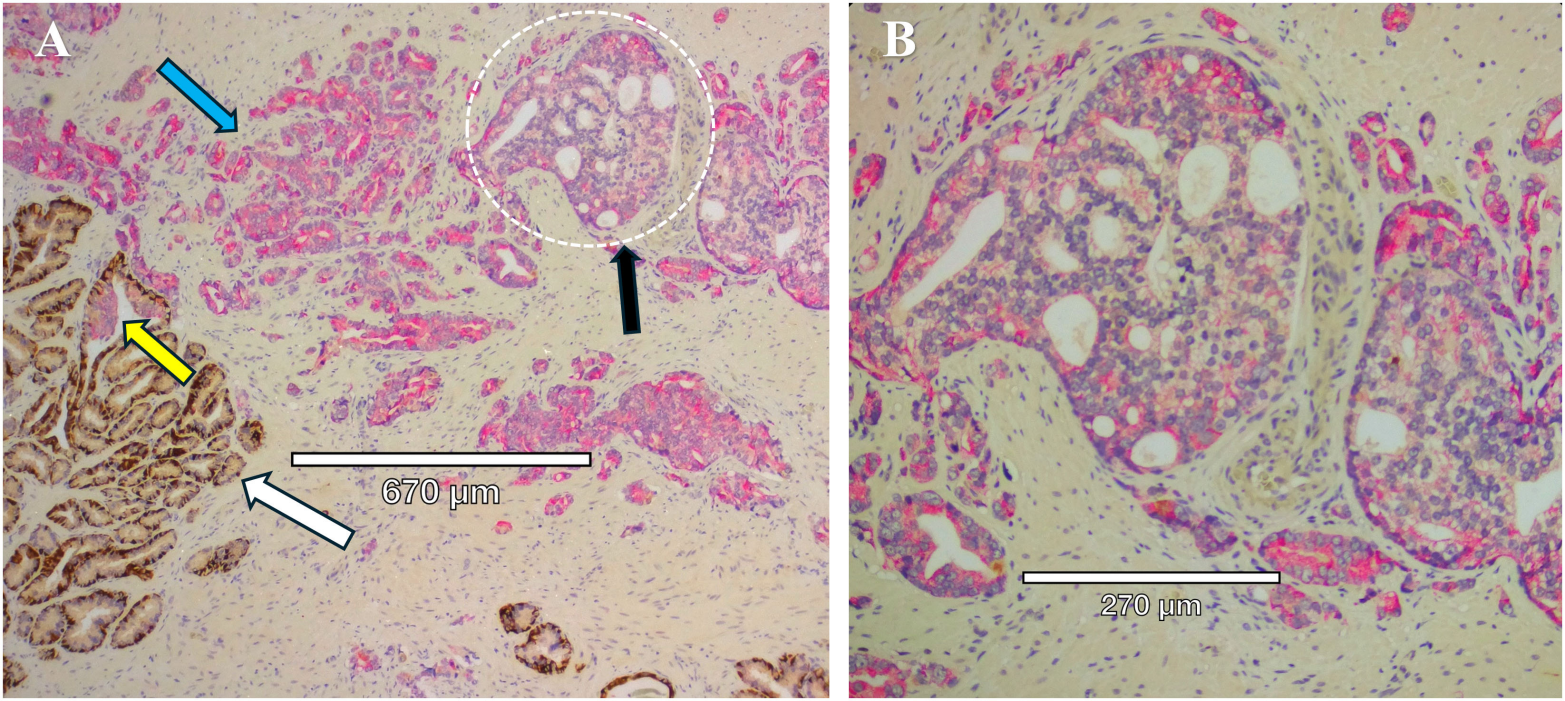
Mixed prostate tumor types in close proximity within the same section. **(A)** Immunohistochemistry staining of prostate tissue obtained from radical prostatectomy to identify HMWCK and p63 (brown) and racemase (pink). White arrow points to normal glands, with intact basal cells identified by HMWCK and p63 (brown). Blue arrow points to Gleason Grade 3 invasive cancer expressing racemase (pink) and lack of basal cells. Black arrow points to a cribriform gland (dotted white circle) and lack of basal cells. Yellow arrow points to a gland with IDC-P, with basal cells mostly present but patchy on the left side of the gland. **(B)** Higher magnification of the cribriform gland, Gleason Grade 4, from panel **(A)**, with characteristic “glands within glands”, variable expression of racemase (pink), enlarged nucleoli, and a high nuclear to cytoplasmic ratio.

**Figure 4:**
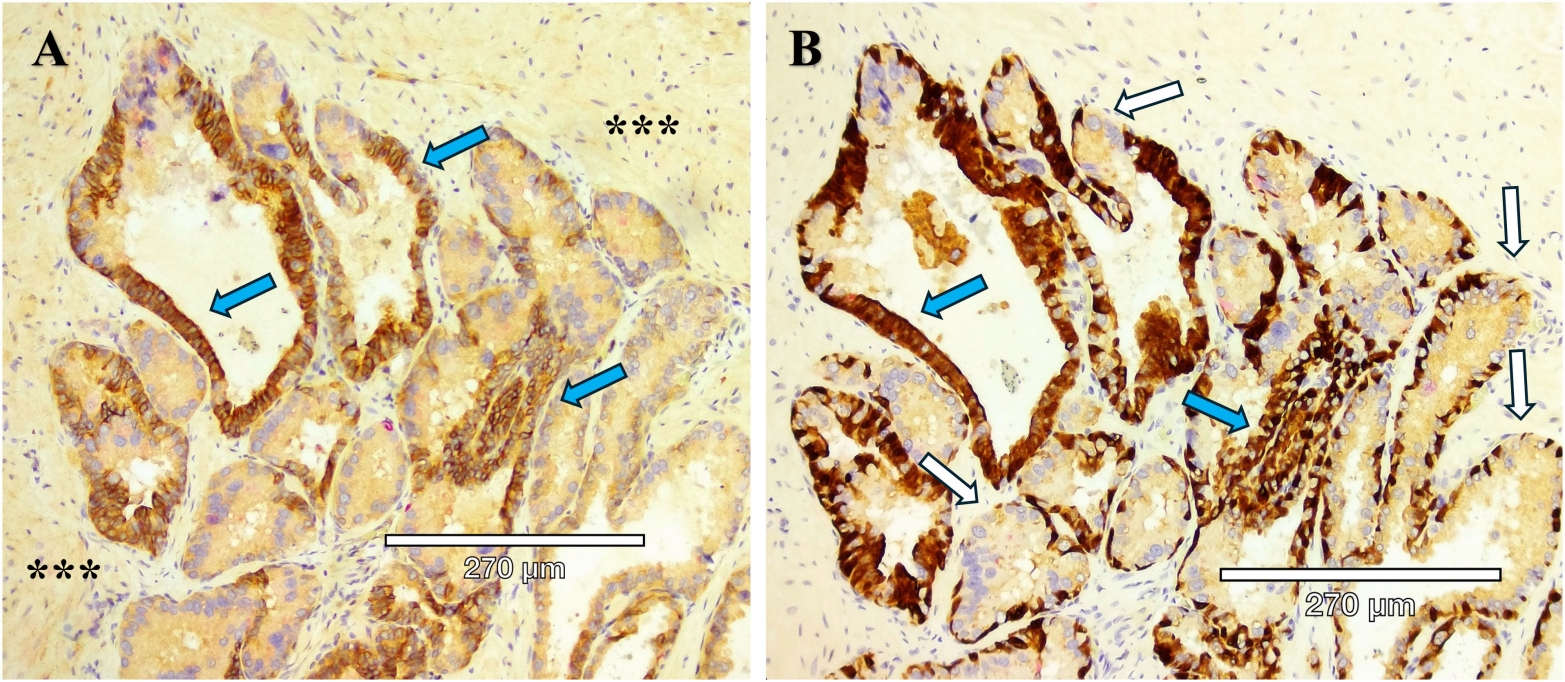
Serial sections of two stains of HG-PIN from a radical prostatectomy sample. **(A)** Kindlin-2 (brown) cytoplasmic stain in an adenocarcinoma, with the expected diffuse kindlin-2 stain in the smooth muscle stroma (***). **(B)** Basal cells (p63, brown) are present except for the budding end of the gland (white arrows), indicative of HG-PIN lesions. A comparison between the serial section staining indicates that kindlin-2 occurs at the cell-cell junctions in basal cells (blue arrows).

Aggressive PCa subtypes are determined by unique histopathological patterns. Normal prostate glands are a simple stratified epithelial bilayer with a layer of luminal cells surrounded by a basal cell layer. The most common lesions identified in PCa patients are adenocarcinomas at 95% of identified tumors, with the other 5% consisting of cribriform architecture (CA, **Figure 1**), intraductal carcinoma of the prostate (IDC-P, **Figure 2**), and prostatic ductal adenocarcinoma (PDA) (23). Often, these phenotypes are characterized by loss of basal cell markers and high racemase staining (**Figure 3**). However, underappreciated aggressive PCa subtypes can display cribriform architecture and intraductal carcinoma. These subtypes are often hypoxic (24, 25), variably express the androgen receptor, and are generally not susceptible to ADT (26). Often these subtypes represent a subpopulation of the tumor and present with normal prostate glands and benign (clear cell cribriform hyperplasia and basal cell hyperplasia), premalignant [high-grade prostatic intraepithelial neoplasia (**Figure 4**)], borderline (atypical intraductal cribriform proliferation), or malignant (intraductal, acinar, ductal, and basal cell carcinoma) lesions.

Cribriform architecture (CA) describes regions of prostate tumors with a confluent sheet of contiguous malignant epithelial cells containing multiple glandular lumens, with no intervening stroma or mucin, which are easily visible at low power, including invasive cribriform cancer (ICC) (**Figure 1**) and as intraductal carcinoma of the prostate (IDC- P) (27) (**Figure 2**). In 2014, the International Society of Urological Pathology (ISUP) declared that cribriform tumors should be categorized within Gleason Grade 4 (28), and in 2019, consensus statements from the ISUP and the Genitourinary Pathology Society (GUPS) recommended reporting cribriform morphology in prostate biopsies and radical prostatectomies (29, 30). Wang et al, has previously shown that CA regions of PCa express high levels of E-cadherin (seen in **Figure 1B**), as well as centrosome amplification, indicative of genomic instability and aggressive disease (3). Still, there has been a tendency to combine IDC-P and CA in risk stratification for intermediate-risk PCa, but as Gordetsky et al, point out, this can be problematic in that some studies have only identified CA as prognostic for biochemical recurrence and increased metastasis, while IDC-P may not show the same predictive value (27). In any case, the clear unambiguous identification of these subtypes is an area of active research that is now within reach due to recent advances in computational pathology.

A unique and highly aggressive form of PCa, intraductal carcinoma of the prostate (IDC-P), is found in about 20% of patients (and as many as 63% of men with advanced disease (26)) and is associated with biochemical recurrence, lymph node metastasis, distant metastasis, increased genomic instability (clustered to genetic regions involved in aggressive PCa) (31), and higher PCa-specific death (32). Kato et al, reported that the 5- and 10-year cancer-specific survival rates in IDC-P-positive patients (with metastatic disease) were 35% and 18%, and those in IDC-P-negative patients were 69% and 53%, respectively (33). Notably, IDC-P can evade ADT treatment and chemotherapy (26, 34, 35). Despite these outcomes, IDC-P has not been a standard element in pathology reports. The GUPS and the ISUP have issued conflicting positions on this issue, with the GUPS rejecting its inclusion in the GG report and the ISUP recommending its inclusion in the GG report (36). Again, this is an opportunity for improved detection and understanding of the molecular events associated with these subtypes to enable a clear understanding of the subtypes and the heterotypic composition of the tumor clusters producing the pro-metastatic phenotype.

## 3 Current detection of intermediate risk prostate cancers

IDC-P is diagnosed based on its unique morphology, consisting of a combination of structural and cytological features (37). IDC-P has two major morphological features: (A) atypical adenocarcinoma cells growing within the pre-existing prostatic glandular structures, and (B) a layer of basal cells that is at least somewhat preserved (37, 38). IDC-P frequently occurs along with cribriform architecture (CA), which is comprised of sheets of cohesive tumor cells with “circular spaces, creating a sieve-like or Swiss cheese appearance,” with the preservation of basal cells surrounding the gland in IDC-P (37, 39). Many studies classify IDC-P and CA together due to the frequency of cribriform growth patterns in IDC-P and the fact that IDC-P frequently coexists with invasive prostate cancer (39, 40). Immunohistochemistry studies have been performed with several markers to indicate the characteristics of the heterotypic prostate cancer subtypes (**Table 3**).

**Table 3:** Common markers of PCa subtypes in intermediate risk prostate tumors.

	HG-PIN	CA	IDC-P	PDA
Incidence	16% of negative biopsies, 80- 100% of PCa (41)	25–34% of RP (42)	23% of PCa (43), ~37% of high-risk disease (44)	2.6% of PCa cases, usually mixed (45), 68% of Mets cases (44)
PTEN (–)	0% (46)	59.3% (47) or 64% (32)	61%–84% (46)	Higher than acinar PCa (48)
P53 (–)	30% (46)	Deletion (31, 49)	60% show deletion (46, 50)	Upregulated (51)
Rb1 (–)	53% (46)	Deletion (31, 49)	81% show deletion (46, 50)	87.5% show deletion (52)
TMPRSS2::ERG fusion	16% (53) or 19% (54) if PCa adjacent	~ 40% (55)	75% (46)	Much less frequent than in PCa (56, 57)
Basal cells (p63)	Intact (49)	Absent (49)	Mostly intact (49)	Absent or patchy (44)
Hypoxia	High (58)	64% (59)	64% (59)	Similar to PCa (60)
PSMA	48.6% (61)	Highly expressed (62)	Low (63)	Low (63)
Ki67	Elevated (64) in luminal cells	Elevated (65)	Similar to PCa (66)	Significantly higher than PCa (52, 57)
EZH2	~ 17% (67)	Increased (42)	~ 46% (68)	98% (69)

The subtypes include high-grade prostatic intraepithelial neoplasia (HG-PIN), cribriform architecture (CA), intraductal carcinoma of the prostate (IDC-P), and prostatic ductal adenocarcinoma (PDA). Percentages denote the percent of cases that were positive for the listed marker. The numbers in parentheses are the citations to the study reporting the data.

In the European Randomized study of Screening for Prostate Cancer (Rotterdam cohort), 79 out of 98 men had classical Gleason score ≤6 prostate cancer (70). Of these, eight of 15 (53%) PCa deaths with classical Gleason score ≤6 were reclassified to modified Gleason score 3 + 4 = 7, contrasted by 16 out of 64 (25%) men with classical Gleason score ≤6 who received modified Gleason score and did not die from PCa. Five out of the eight (63%) men with fatal prostate cancer whose Gleason score was modified up had IDC-P/CA, compared with only two out of 16 (13%) Gleason score-reclassified men with non-fatal PCa (P = 0.011) (70).

CA and IDC-P frequently co-occur, with 47% of CA occurring with IDC-P and 68% of IDC-P occurring with CA (42, 71). However, it is also important to note that while IDC-P often presents with cribriform architecture, invasive cribriform carcinoma (ICC) is not the same as IDC-P (27, 42). While there are many similarities between IDC-P and CA, there are distinct differences, most notably the preserved presence of basal cells in IDC-P but not in CA/ICC (**Table 3**). Interestingly, while PTEN loss is common in both CA and IDC-P (more so in IDC-P), it does not appear to be the primary driver for poor outcomes in cribriform tumors (72). Increased hypoxia levels, a known driver of tumor growth and escape from the gland (25, 73), are noted in both CA and IDC-P (59). Earlier research suggested the presence of BRCA2 with IDC-P (74, 75), which prompted the NCCN to add a Genetic/Familial High-Risk Assessment that would include germline testing in men who have PCa with CA or IDC-P (49). In a more recent study, however, Lozano et al, found no association between germline BRCA2 (gBRCA2) mutations and cribriform/IDC-P histology in primary prostate tumor samples (49, 76). Although there was a slightly higher rate of gBRCA2 in patients with CA (53% gBRCA2 vs 43% non-carriers), there was a lower rate in IDC-P patients (36% gBRCA2 vs 50% non-carriers) (76). These results suggest a need to refine the current guidelines and indicate that further investigation is needed into the role of germline BRCA2 alterations in CA/IDC-P phenotypes of PCa (49).

Wong et al. (42), offer one of the few analyses of the tumor microenvironment (TME) for CA/IDC-P, finding that they both are associated with an altered TME that leads to immunosuppression and that this TME feature prevents an effective immune response. Further, this study found that benign epithelial cells in the CA/IDC-P TME presented with differential gene expression reflecting amplified inflammatory response and signaling compared to the same cell types in a benign prostate environment (42). These results suggest that even non-tumor cells in the prostate microenvironment are recruited to sustain and promote tumor growth. The authors also identified a CA/IDC-P immunosuppressive cancer-associated fibroblast (CAF) gene signature based on four upregulated genes (CTHRC1, ASPN, FAP, and ENG), which they termed “CAFÉ CAF,” and which is associated with adverse outcomes (42)— worse PCa progression-free survival in The Cancer Genome Atlas (TCGA) PanCancer Atlas prostate adenocarcinoma cohort (77) and worse disease-free survival in the Memorial Sloan Kettering Cancer Center (MSKCC) prostate adenocarcinoma cohort (42, 78). Along with the immunosuppressive functions of CAFs, the research indicates that antitumorigenic immune cells were suppressed while pro-tumorigenic immune cells were enriched in the CA/IDC-P TME (42).

## 4 Biopsy vs. prostatectomy for CA and IDC-P

Considerable research suggests that there is a strong tendency toward false negatives for CA and IDC-P in biopsy samples. In a study of 836 patients who underwent radical prostatectomy (RP), 26% of the patients had a false-negative biopsy (79). Another study by this group found, in a group of 287 radical prostatectomy samples, 241 (84%) had cribriform morphology and 161 (56%) had IDC-P, suggesting that the sensitivity of biopsy (RP as the reference) was 42.4% for IDC-P and the biopsy sensitivity for detection of either IDC-P or CA was 52.5% (80). In the same study the authors found that, among men who had multiparametric magnetic resonance imaging–guided biopsies (mpMRI), the sensitivity was 54% for discovery of CA and 37% for discovery of IDC-P (80). However, there is some debate about the accuracy of mpMRI in identifying PNI or CA (81). Others have found that apparent diffusion coefficient (ADC) imaging reconstruction is more sensitive in identifying CA, especially when there is only one identified tumor lesion (82). In a large longitudinal study of more than 10,000 men, 1 in 12 patients given a Gleason Grade Group 1 diagnosis based on non-targeted biopsy were later found to harbor aggressive disease upon radical prostatectomy (83). In another study, Ericson et al, found that the sensitivity of biopsy to detect CA/IDC-P at prostatectomy was 56.5% while specificity was 87.2%, and that among 273 patients with active surveillance eligible tumors (NCCN very low, low, and favorable intermediate risk) sensitivity was 34.4% and specificity was 88.1% (84). These results indicate that biopsy has low sensitivity for detecting CA and IDC-P, and clinical decision-making must take these limitations into account, especially in determining which low-risk cancers can be assigned to active surveillance. As Bernardino et al, suggest, biomarkers for better detection of these histological patterns are needed (80).

While biopsy sensitivity is a serious problem, a 2019 survey revealed that only 40% of U.S. GUPS pathologists reported the presence of cribriform glands on patient biopsies (30), making an accurate treatment strategy impossible in those cases where CA is not recorded (**Figure 3**). Importantly, Bernardino et al, found that the presence of IDC-P predicts lymphatic metastasis—of 52 patients who displayed evidence of metastasis, 41 (79%) exhibited indications of lymphatic metastasis (43). In patients with biochemical recurrence (BCR) and metastatic disease detected via PSMA PET/CT, the presence of CA is associated with metastasis to lymph nodes (85).

## 5 Prostatic ductal adenocarcinoma

A lesser known and statistically rare (0.4% to 0.8% of all prostate cancers (86)) form of prostate cancer is ductal adenocarcinoma of the prostate (23, 48, 87). While acinar adenocarcinoma of the prostate accounts for 95% of prostate cancer cases, of the remaining 5%, prostatic ductal adenocarcinoma (PDA), intraductal carcinoma of the prostate (IDC-P), and cribriform architecture (CA), are the most common subtypes (23). First described in 1967 by Melicow and Pachter (88), there is not yet an agreed upon nomenclature for this PCa subtype. Recently, ductal adenocarcinoma (DAC) (48), prostatic ductal adenocarcinoma (PDA) (23), and ductal prostate cancer (dPC) (87) have been used to describe what was once known as “endometrioid” or “papillary” carcinoma (48). Most ductal adenocarcinomas arise in the peripheral zone, but a small subset develops in the transition zone around the prostatic urethra (89). In a meta-study of 2,907,170 prostate cancer cases, of which 5911 were PDA, this subtype was more likely to present as T3 and T4 stages, with far greater occurrence of metastatic disease compared to typical prostate cancer (48). Interestingly, PDA metastasized to unusual sites, including penis (48), lung, and liver (86, 90), rather than the bone metastases commonly seen in prostatic adenocarcinoma (91). As is found in IDC-P, PDA also shows an underlying upregulation of androgen-resistance pathways, making it less amenable to conventional ADT and similar treatments (92, 93). Shi et al, reported a single-center retrospective study where 93.7% of patients with metastatic PDA were originally treated with standard ADT, yet around 85.8% experienced disease progression after the initial treatment (23, 94).

There are three basic presentation patterns for PDA: papillary (78.7%), cribriform (14.7%), and PIN-like (6.6%) (60). While only 14.7% of the tumors displayed the cribriform phenotype in that study, 67.2% of the cases harbored high grade GS ≥8 disease and 27.9% displayed comedonecrosis, a feature of highly aggressive PCa (60). PDA generally displays papillary and large cribriform growth patterns, and the papillae often have fibrovascular cores, while the cribriform glands have slit-like slender lumens (89). PDA is present in ~2.6% of cases and is usually mixed with acinar adenocarcinoma (45). PDA in the absence of acinar adenocarcinoma accounts for less than 1% of prostate cancers (48, 95). In RPs, the term “ductal adenocarcinoma” is used “arbitrarily” for those tumors with >50% ductal morphology, while in biopsies, the term “adenocarcinoma with ductal features” is recommended even when it shows a pure ductal pattern (89). While there are morphologic and phenotypic differences between ductal and acinar carcinoma, there are few molecular differences between the two (92, 96). The 5th edition of the World Health Organization (WHO) Classification of Urinary and Genital Tumors recommends that all PDAs be assigned Gleason grade 4, with the exception of those with comedonecrosis, which are considered to represent Gleason grade 5 (89) and PIN-like PDA, which is assigned a Gleason grade 3 (92).

## 6 Androgen-indifferent prostate cancer

Androgen independence can arise in PCa subtypes within the primary tumor that are indifferent to ADT or other androgen receptor signaling inhibitors, thus mimicking castration resistance (97). Fiñones et al, isolated and propagated androgen-independent cells from prostatectomy samples of early, localized (Stage-I) cases, grew them as spheroids, and then xenografted 22 of these as PDXs in intact and castrated SCID mice, generating histologically typical locally-invasive human PCa or undifferentiated cancers that lacked PSA expression (98). The authors report that the propagation of stem/progenitor-like castrate-resistant PCa cells derived from early human prostate carcinomas suggests there is a subpopulation of cells resistant to androgen-deprivation therapy and which may drive the subsequent emergence of disseminated androgen-indifferent PCa (98). In a large transcriptomic study of AR expression in treatment-naive primary PCa, a unique subgroup of low AR activity tumors was found in approximately 10% of samples (97, 99). The low AR activity subgroup was enriched in higher-grade tumors with reduced sensitivity to ADT and greater sensitivity to platinum chemotherapy (97, 99). Hamid et al, analyzed samples from 43 patients with *de novo* metastatic and 205 localized hormone-naive prostate cancers and found combined (two or more) alterations in the tumor suppressors TP53, RB1, and PTEN in 28% and 11% of cases, respectively, a finding associated with a poor prognosis (97, 100). Androgen-indifferent “subclones” may pre-exist in some primary, untreated tumors and appear only under the selective pressure created by AR inhibition, while in other cases, mutations and/or other molecular alterations may be acquired during therapy-induced castrate-resistant progression that results in androgen indifference (97). Biomarkers that can be monitored repeatedly over time will be crucial in order to identify the emergence of androgen indifference and alter therapeutic decisions to target these castration resistant sub-clones (97).

Early reports have been inconclusive on the function of the androgen receptor in cribriform architecture prostate cancers (101), although it has been reported that IDC-P contains androgen-indifferent cells as part of its heterogeneity (26). In a mouse PDX model, Porter et al, found that IDC-P tumors persisted in 5 of 7 animals treated via castration to mimic ADT, although with lower Ki-67, ERG, and PSA expression, while maintaining AMACR and p63 expression and with the androgen receptor primarily localized to the cytoplasm instead of the nucleus, consistent with the near elimination of systemic androgens (26). The presence of CA at biopsy has been associated with resistance to ADT and ARSi therapies with locally high-risk PCa, resulting in a worse response to therapy (49). Chen et al, identified a specific expression pattern with high levels of nuclear receptor interaction protein (NRIP) and AR, together with a low level of DNA damage binding protein 2 (DDB2) that was found more frequently in PCa with a cribriform pattern than in non-cribriform tumors, suggesting that disturbance of the balance between NRIP and DDB2 may change AR homeostasis and contribute to tumor aggressiveness in certain subtypes of prostate cancer (102). Taken together, these studies suggest that these intermediate-risk PCa subtypes can contain androgen-indifferent populations and may require a different treatment approach.

## 7 A case report illustrates the challenge of mixed tumor phenotypes

McDonald et al, reported on a single case recently for the Journal of Clinical Pathology (103), which is summarized here. The patient presented with a PSA of 5.1 ng/mL and a multiparametric MRI (mpMRI) demonstrating a Prostate Imaging Reporting & Data System (PI-RADS) 5 lesion in the right peripheral zone at the mid-gland and apex. Following a trans-perineal prostate biopsy with targeted and standard template cores, it was found that the patient had IDC-P (dense cribriform architecture) in the target zone and throughout the right lobe. Low-volume Gleason score 3 + 3 = 6 invasive adenocarcinoma and IDC-P were found contralaterally to the PI-RADS 5 lesion, in the left posterior zone. In the left middle and anterior zones, neither IDC-P nor invasive carcinoma was detected. The issue the authors address in their report is the confusion that arises with a mixed phenotype diagnosis and differing reporting criteria between the ISUP and GUPS.

Grading using GUPS offered a Gleason score of 3 + 3 = 6 and corresponding GG1. Because the GUPS does not include IDC-P in the Gleason and GG scoring, only the 3 + 3 = 6 lesion was included in the final grade. However, grading using ISUP criteria offered a Gleason score of 4 + 5 = 9 and corresponding GG5, because the concurrent high-volume IDC-P was included in the final grade. The patient subsequently underwent a prostate-specific membrane antigen positron emission tomography (PSMA-PET) scan that suggested agreement with the PI-RADS 5 lesion diagnosis on mpMRI, and no indication of nodal or distant disease (103).

Final histology after prostatectomy demonstrated right-sided acinar (95%) and ductal (5%) adenocarcinoma with widespread IDC-P, ECE, and invasion into the right seminal vesicle. The Gleason score was 5 + 4 = 9 and GG5. 80% of the carcinoma was intraductal. There was a separate small tumor in the left posterolateral mid-zone, Gleason score 3 + 3 = 6, GG1, corresponding to the GG1 biopsy finding (103). Under the GUPS guidelines this patient would have been recommended for active surveillance, while under the ISUP guidelines, this patient would have received aggressive treatment commensurate with a GG5 tumor burden. The authors mention a study by Khani and Epstein (104), in which 18% of patients with low-grade PCa and concurrent IDC-P were incorrectly offered active surveillance due to IDC-P not being factored into the Gleason score and its significance not being understood (103). The GUPS guidelines must be brought into alignment with the ISUP guidelines, and we must find better clinical markers for unfavorable intermediate risk PCa.

## 8 Clinical prognosis for three of the subtypes

Outcomes are not good for patients who harbor these aggressive phenotypes alongside favorable intermediate risk prostate adenocarcinoma. Standard pathology practice suggests that when these phenotypes are present and are not the dominant tumor type, their Gleason score is secondary to the primary acinar carcinoma, for example 3 + 4, and while this would typically be a score eligible for active surveillance, the aggressiveness of the secondary tumor phenotype increases the aggressiveness of the cancer and requires a more intensive treatment strategy.

### 8.1 Prostatic ductal carcinoma

The overall survival (OS) rate for PDA is 67% at 5 years and a disease-free survival (DFS) at 5 years of 34% (105), compared to nearly 99% for local or regional PCa (106). Further, PDA is more likely to present as T3 stage compared to PCa—on meta-analysis, the percentage of T3 disease is 22.2% in PDA and 8.9% in PCa (48). The presence of PDA at initial needle biopsy is associated with a higher risk of biochemical recurrence (BCR) following definitive treatment (RP) and an increased risk of progression to metastases, with a 5-year metastasis-free survival rate of 75% (versus 95% for PCa) (93). Ranasinghe et al, conducted a genomic analysis of PDA that found 10/11 (91%) PDA tumors treated with ADT had upregulation of androgen-resistant pathways, and that none of the PDA patients (0/15) who received only neoadjuvant ADT prior to RP had any pathologic downgrading (93).

### 8.2 Intraductal carcinoma of the prostate

Patients with IDC-P at biopsy or RP are more likely to have higher Gleason grade PCa, more advanced pathological stage, and more serious clinical features, such as extraprostatic extension and regional lymph node involvement (107). Patients with IDC-P at prostatectomy have reduced time to BCR and reduced progression-free survival (PFS) (107–111), even after neoadjuvant hormonal therapy or chemotherapy (35, 107, 108, 112). Among distinct patient groups, IDC-P is also associated with poorer cancer-specific survival and OS (107, 108, 113–115), and IDC-P is correlated with decreased survival in patients who have already progressed to metastatic disease, suggesting that it indicates worse clinical outcomes regardless of disease stage (34, 107, 116, 117). While some early research suggested that IDC-P was a transitional stage of HG-PIN becoming PCa (20), research by Zhao et al, identified three forms of IDC-P: early divergent (71%), late divergent (29%), and clonally distant (23%) (118), with the late divergent subtype presenting with a higher fraction of shared somatic alterations between the concurrent IDC- P and PCa compared with those of the early divergent pattern. Further, Zhao et al, found that only one patient (1/5) in the late divergent evolutionary pattern progressed within 24 months, while 5/12 patients (41.7%) and 3/5 patients (60.0%) progressed in the early divergent and clonally distant patterns, respectively (118).

### 8.3 Cribriform architecture

In a meta-analysis of studies evaluating clinical outcomes in PCa with cribriform architecture, Russo et al, found that CA was associated with higher risk of ECE (odds ratio [OR] 1.96), seminal vesicle invasion (SVI) (OR: 2.89), and positive surgical margins (PSM) (OR: 1.88); they additionally showed that CA was associated with greater risk of BCR (hazard ratio [HR]: 2.14) and of cancer-specific mortality (CSM) (HR: 3.30) (119). Sayan et al, evaluated cribriform architecture (Gleason 4) in 394 patients, 129 (32.74%) of which had cribriform patterns (120). Among those patients with CA versus those without CA, there was a longer median follow-up [46.37 months vs 37.27 months], a higher pre-RP prostate specific antigen (PSA) score [8.10 ng/mL vs. 7.00 ng/mL], a higher Gleason score (52% versus 27%), and they were more likely to have T3a or higher stage PC (76% versus 53%) (120). Perhaps more importantly, Sayan’s group identified seven “hub genes” (KRT13, KRT5, KRT15, COL17A1, KRT14, KRT16, and TP63), four of which are basal cell specific, that demonstrated notably lower mRNA expression levels in patients with CA compared to those without (120). Clinically, the recognition of this gene set may allow for easier identification of a subset of patients with unfavorable histopathological characteristics who are at a higher risk of reduced PFS and have unique genomic alterations (120). Other researchers have identified long noncoding RNA SChLAP1 as a predictor of biochemical recurrence in PCa, and its presence is associated with adverse clinicopathological characteristics, including higher GG, higher pT stage, invasive CA/IDC-P, and reactive stroma (121). However, SChLAP1 is very heterogeneous, and the authors suggest there must be high levels in multiple biopsy samples to be definitive (121).

Robert Bristow’s group identified genomic instability, the presence of SChLAP1, and hypoxia as the “nimbosus” which leads to increased metastatic capability and lethality (59). While hypoxia is a central feature of aggressive PCa, and is associated with CA in particular (122), further exploration of that topic is beyond the scope of this review. Finally, in a group of 16 patients with GG1 PCa without diagnosed CA at biopsy, 14 (87%) showed CA or IDC-P at RP, with 12 showing CA (123). These results highlight the need for more precise diagnostic markers to detect CA in PCa at the time of biopsy.

## 9 Future approaches in computational histopathology

At present, there are no clear patterns in identifying the extent and aggressiveness of CA and IDC-P prior to radical prostatectomy using FDA-approved imaging techniques, tumor morphology, local invasion patterns, genetics, or available biomarkers. On the other hand, there is increasing development of artificial intelligence (AI) driven computational and combinatorial techniques that may offer more sensitive and specific diagnostic capabilities.

Prostate cancer detection and grading using objective criteria is an ongoing challenge. Recent advances in generative AI technology suggest that tissue characteristics learned by machine-based algorithms will coincide with diagnostic features used by pathologists. For example, a framework that enforces algorithms to learn the cellular and subcellular differences between benign and cancerous prostate glands in digital slides from hematoxylin and eosin-stained (H&E) tissue sections is now possible [reviewed in (124)]. After accurate gland segmentation and exclusion of the stroma, the central component of the pipeline, named HistoEM, utilizes a histogram embedding of features from the latent space of the convolution neural networks encoder (125). This approach allows for computer-learned features to be visualized and could be applied to morphologically distinct tumors of the cribriform type.

There have also been breakthroughs in virtual staining of a single H&E slide for multiplexed tissue markers. The Virtual Immunohistochemistry Multiplex staining (VIMs) model is designed to generate multiple immunohistochemistry (IHC) stains from a single H&E-stained tissue section. IHC stains are a central feature of pathology practice for clarifying complex diagnostic questions and generating appropriate patient treatment decisions (126). There are many advances occurring in the use of AI for image analysis applications that are being leveraged for PCa diagnosis and risk stratification, and these can be trained to identify cribriform and IDC-P subtypes of PCa. Interested readers are referred to a recent and comprehensive review published by a team headed by Lawrence True (127).

In addition, the development and use of multiplexing of relevant cribriform biomarkers would likely generate new objective criteria to associate with aggressive outcomes. For example, a recent multicenter study reported on a pipeline for evaluation of machine learning/artificial intelligence models to quantify programmed death ligand 1 (PD-L1) immunohistochemistry (IHC). Transparent and stepwise performance metrics can be applied to any IHC assay to evaluate commercial automated IHC scoring systems using any new cribriform biomarkers (128). The continued improvement of the biomarkers along with the increased ability to analyze IHC images will add significantly to management choices for cribriform type prostate cancer.

## 10 Discussion

The goal of this review was to highlight the existence of aggressive tumor subtypes in intermediate risk prostate cancer. While we have tried to be comprehensive in this focused area of our review, we recognize limitations due to the needs of a brief review. However, we have highlighted the diagnostic and clinical challenges of these subtypes, from poor reliability and specificity in biopsy and imaging (80) to the challenges of treating tumor types that are resistant to the standard-of-care (26).

The most well-documented aggressive subtypes are intraductal carcinoma of the prostate and tumors with cribriform architecture, both of which are associated with decreased time to BCR, increased presence of ECE, increased metastatic potential, and shorter cancer-specific mortality (129). While there is considerable evidence that these subtypes are more metastatic and often overlap, there is disagreement among the two main urology groups (ISUP and GUPS) as to whether or not these subtypes should be recorded on the pathology report and, if so, what grade they should be given (130). Both groups recommend that isolated IDC-P (no adenocarcinoma present) should not be graded; however this is a relatively rare occurrence, being reported in 0.006-0.26% of prostate needle biopsies (131). In the presence of prostatic adenocarcinoma, the ISUP recommends incorporating the IDC-P component of invasive prostate cancer in the Gleason score, whereas the GUPS recommends reporting IDC-P as a comment, independent of the Gleason score (130). Both the ISUP and the GUPS recommend recording cribriform tumor patterns in the pathology reports as Gleason score 4 (132).

The majority of these PCa subtypes arise in hypoxic regions of the gland, as is true with most variations of prostate cancer (133). Hypoxia in the prostate is associated with increased chromosomal instability and gene amplification, downregulation of DNA damage repair pathways, and altered vulnerability to DNA-damaging agents (133). However, there is very little consensus on the cellular and molecular origins of prostate cancer, other than the agreement that only ~12% of PCa is genetically inherited, primarily in DNA damage repair genes, such as BRCA2 (134). In addition, there is little agreement on the origins of CA and IDC-P, other than the previously mentioned association with increased genetic instability, copy number alterations, and genetic alterations in a handful of genes, including BRCA2, TP53, RB1, PTEN, and a few others (42), but the usefulness of the BRCA2 mutation in CA/IDC-P recently has been questioned (76). It is our hope that future research, possibly using the power of AI, will likely identify better and more accurate biomarkers to increase the effectiveness in identifying CA and IDC-P in their early stages, before they can become metastatic.

## 11 Conclusion

While PCa is most often an indolent disease that men will die with and not from, there remains an unmet clinical need to identify, at the localized stage of the cancer, which tumors are aggressive and will progress into metastatic disease. Currently, both biopsy and various forms of imaging are not capable of high specificity or sensitivity in identifying aggressive subtypes of PCa while the tumor is still organ-confined. The increased use of AI technologies for imaging and diagnosis may offer new biomarkers or IHC imaging strategies that increase the diagnostic accuracy of these potentially lethal subtypes of PCa.
